# Adipose Derived Stem Cells Affect *miR-145* and *p53* Expressions of Co-Cultured Hematopoietic Stem Cells

**DOI:** 10.22074/cellj.2018.4393

**Published:** 2017-11-04

**Authors:** Tahereh Foroutan, Aisan Farhadi, Saeed Abroun, Bahram Mohammad Soltani

**Affiliations:** 1 Department of Animal Biology, Faculty of Biological Sciences, Kharazmi University, Tehran, Iran; 2 Department of Hematology, Faculty of Medical Sciences, Tarbiat Modares University, Tehran, Iran; 3 Department of Genetic, Faculty of Biological Sciences, Tarbiat Modares University, Tehran, Iran

**Keywords:** Adipose Cell, Hematopoietic Stem Cell, MicroRNA

## Abstract

**Objective:**

Umbilical cord blood is used for transplantation purposes in regenerative medicine of hematological disorders. MicroRNAs are important regulators of gene expression that control both physiological and pathological processes such as cancer development and incidence. There is a new relation between *p53* (tumor suppressor gene) and *miR-145* (suppressor of cell growth) upregulation. In this study, we have assessed how adipose-derived stem cells (ADSCs) affect the expansion of hematopoietic stem cells (HSCs), as well as *miR-145* and *p53* expressions.

**Materials and Methods:**

In this experimental study, we cultured passage-3 isolated human ADSCs as a feeder layer. Flow cytometry analysis confirmed the presence of ADSC surface markers CD73, CD90, CD105. *Ex vivo* cultures of cordblood CD34^+^cells were cultured under the following 4 culture conditions for 7 days: i. Medium only supplemented with cytokines, ii. Culture on an ADSCs feeder layer, iii. Indirect culture on an ADSCs feeder layer (Thin Cert™ plate with a 0.4 µm pore size), and iv. Control group analyzed immediately after extraction. Real-time polymerase chain reaction (PCR) was used to determine the expressions of the *p53* and *miR-145* genes. Flow cytometry analysis of cells stained by annexin V and propidium iodide (PI) was performed to detect the rate of apoptosis in the expanded cells.

**Results:**

ADSCs tested positive for mesenchymal stem cell (MSC) markers CD105, CD90, and CD73, and negative for HSC markers CD34 and CD45. Our data demonstrated the differentiation potential of ASCs to osteoblasts by alizarin red and alkaline phosphatase staining. MTT assay results showed a higher proliferation rate of CD34^+^cells directly cultured on the ADSCs feeder layer group compared to the other groups. Direct contact between HSCs and the feeder layer was prevented by a microporous membrane *p53* expression increased in the HSCs group with indirect contact of the feeder layer compared to direct contact of the feeder layer. *p53* significantly downregulated in HSCs cultured on ADSCs, whereas *miR-145* significantly upregulated in HSCs cultured on ADSCs.

**Conclusion:**

ADSCs might increase HSCs proliferation and self-renewal through miR-145, p53, and their relationship.

## Introduction

Umbilical cord blood is used for transplantation in regenerative medicine for hematological disorders. Improvement of hematopoietic reconstitution and engraftment potential of *ex vivo*-expanded hematopoietic stem cells has been unsuccessful due to the inability to generate an adequate amount of stem cells. Many studies report that control of *in vitro* hematopoietic stem cell (HSC) self-renewal is difficult. Hematopoietic cytokines fail to support reliable amplification of *in vitro* HSCs and additional factors appear to be needed (1). Recently, factors such as feeder layers are suggested to affect HSCs expansion (2). Expanded HSCs derived from cord blood cultured on a feeder layer of mesenchymal stem cells (MSCs) have reduced apoptosis rates (3). Adiposederived stem cells (ADSCs) show properties similar to that observed in bone marrow MSCs. Because of the ease of accessibility human, researchers consider ADSCs to be an attractive source for regenerative medicine (4). ADSCs are immunoprivileged, prevent severe graft-versus-host disease, and stable in culture (5). ADSCs show high intrinsic expression of self-renewal factors compared to bone marrow-derived MSCs (2). In the current study, we have used ADSCs as a feeder layer for HSC expansion because they produce various factors to support stem cell maintenance and cell growth.

MicroRNAs, a large group of negative gene regulators, work through a post-transcriptional suppression mechanism. MicroRNAs play an important role in proliferation, differentiation, and apoptosis (6). They are short, noncoding RNAs, usually 18-25 nucleotides in length, which repress translation and cleave mRNA by base pairing to the 3′untranslated region of the target genes (7). Although various numbers of microRNAs have been studied in HSCs, there are few reports that pertain to the function of *miR-145*. Human *miR-145* is broadly expressed in germline and mesoderm-derived tissues such as the breast (8), ovaries (9), testes, uterus, prostate, heart, and spleen (6). Sachdeva and Mo (6) have reported *miR-145* mediated suppression of cell growth, invasion, and metastasis. Based on these findings, they proposed that as a tumor suppressor, *miR-145* might be a valuable biomarker for cancer diagnosis. Starczynowski et al. (7) reported that deletion of chromosome 5q in patients with 5-q32-33 syndrome correlated with the loss of *miR-145* and *miR-146a*, two microRNAs frequently observed in HSCs. It was reported that in various cancers, *miR-145* prevents tumor angiogenesis and metastasis by targeting c-Myc (10, 11). In the present research, we have investigated the expression levels of *p53* and *miR-145* in HSCs after culture on feeder layers of ASDCs. It is well known that *p53* upregulates *miR-145* expression (12). Previous studies have shown the transcriptional induction of miR-145 by *p53* in response to anticancer drugs or serum starvation. *p53* induces expression of tumor suppressor *miR- 145* (13, 14). In this study, we investigated the expression levels of *p53* and *miR-145* in HSCs after culture on a feeder layer of ADSCs.

## Materials and Methods

### Adipose-derived stem cell culture

We obtained human subcutaneous adipose tissue samples from donors who underwent abdominoplasty in Erfan Hospital Iran). The patient gave consent to use of donated samples in the present study. The tissue samples were processed according to a modified procedure by Zuk et al. (15), which included 0.075% collagenase II (Sigma-Aldrich, St. Louis, MO) for 30 minutes, followed by centrifugation at 150 g for 5 minutes. The pellet was washed three times in phosphate buffered saline (PBS, Gibco, Germany), then we seeded the cells at 10^5^ cells/dish and cultured them in Dulbecco’s modified eagle’s medium (DMEM, Gibco, Germany), 10% fetal bovine serum (FBS, Gibco, Germany), and 100 U/ml penicillin/streptomycin. Human HSCs were obtained from Royan Institute. The Institutional Review Board and Ethical Committee of Royan approved the HSCs extraction method.

### Proliferation and phenotype analyses

We used flow cytometry to detect ADSCs surface markers monoclonal antibodies were used for CD73, CD90, and CD105 markers. To enable differentiation into osteoblast cells, we used passage-4 ADSCs and a medium that consisted of high glucose DMEM, 10% FBS, 10 nM dexamethasone (Sigma-Aldrich, USA), 35 mg/mL of ascorbic acid, and 1 mM β-glycerophosphate (Chemicon, USA). Cells were incubated in 5% CO_2_ at 37˚C for 21 days. We used alizarin red to confirm differentiation into osteoblast cells. An alkaline phosphatase kit (Sigma- Aldrich) was used for alkaline phosphatase activity.

### CD34^+^ cell isolation and culture (group design)

Mononuclear cells were separated with Ficoll (1.077 ± 0.001 kg/L, Sigma-Aldrich, USA). Next, we incubated these cells with anti-CD34 antibody labeled with Fe nanoparticles (America Milton Biotech), after which CD34^+^ cells were separated by manual cell separation using a MACS column (America Milton Biotech). Anti- CD34 were used to confirm the CD34 marker in isolated cells obtained from umbilical cord blood. After feeder layer preparation with mitomycin C the CD34^+^ cells were cultured under the following 4 culture conditions for 7 days: i. Stem span medium only supplemented with 100 ng/ml of the following cytokines: stem cell factor (SCF), thrombopoietin (TPO), and fetal liver tyrosine kinase 3 ligand (Flt-3L), ii. Direct culture on an ADSCs feeder layer, iii. Indirect culture on an ADSCs feeder layer (ThinCert™ plate with a 0.4 μm pore size), and iv. Control group of cells analyzed immediately after extraction.

### MTT assay

The 3-(4, 5-dimethylthiazol-2-yl)-2, 5-diphenyltetrazolium bromide (MTT) assay was used to assess cell viability for all groups. This assay measures the amount or ratio of cell proliferation. It is a colorimetric assaydependent on the reduction of the tetrazolium salt, MTT, to form blue formazan crystals. After incubation, we removed the overlying culture medium and added MTT. Next, the cells were incubated for 4 hours in an incubator in CO_2_ at 37˚C. Isopropanol acid was added and we read the optical density (OD) of the obtained solution at 630nm as the reference wavelength and 570 nm as the measurement wavelength using the ELISA reader. Oneway ANOVA was used for data analysis.

### Annexin V evaluation of apoptotic cells

We used an Apoptosis kit (Bioscience, USA) for apoptosis analysis. At culture day 14, we treated 1×10^4^ cells resuspended in 1x binding buffer with fluorochromeconjugated annexin V for 10 minutes. Next, cells were washed and resuspended in 1x binding buffer. A propidium iodide (PI) solution was added and fluorescence of the stained cells was analyzed by flow cytometry.

### Reverse transcription and real-time polymerase chain reaction

RNA was extracted from sample cells using TRIzol (Fermentas, Germany). The cDNA was synthesized using a cDNA synthesis kit (Fermentas, Germany) based on the manufacturer’s instructions.Primers were designed according to the NCBI website and synthesized by Bioneer Company. SYBER green master mix was used for the polymerase chain reaction (PCR) reactions (Applied Biosystems, USA). The real time quantitative (qRT) PCR program was performed with a melting cycle for 5 minutes at 95˚C followed by 10 seconds at 95˚C, 40 cycles of melting, 15 seconds at 60˚C (annealing), and 30 seconds at 72˚C (extension). The sequences for *GAPDH, p53,* and *MiR-145* are as follows:

*p53*: F: 5′-TCCTCAGCATCTTATCCGAGTG-3́
  R: 5́-AGGACAGGCACAAACACGCACC-3 ´
*GAPDH*: F: 5′-ATGGGGAAGGTGAAGGTCG-3 ´
  R: 5 ´-GGGGTCATTGATGGCAACAATA-3 ´
miR-145**: F: 5′-GTCCAGTTTCCCABGGAA-3′
  R: 5́-TGACCCCAGGTAACTCTGAGTGT-3 ´

### Statistical analysis

Data are presented as mean standard deviation (SD). We used the two-way ANOVA and Duncan test for data analysis. Differences were considered significant at P<0.05. In the present study, all experiments were repeated 3 times.

## Results

**Fig.1:**
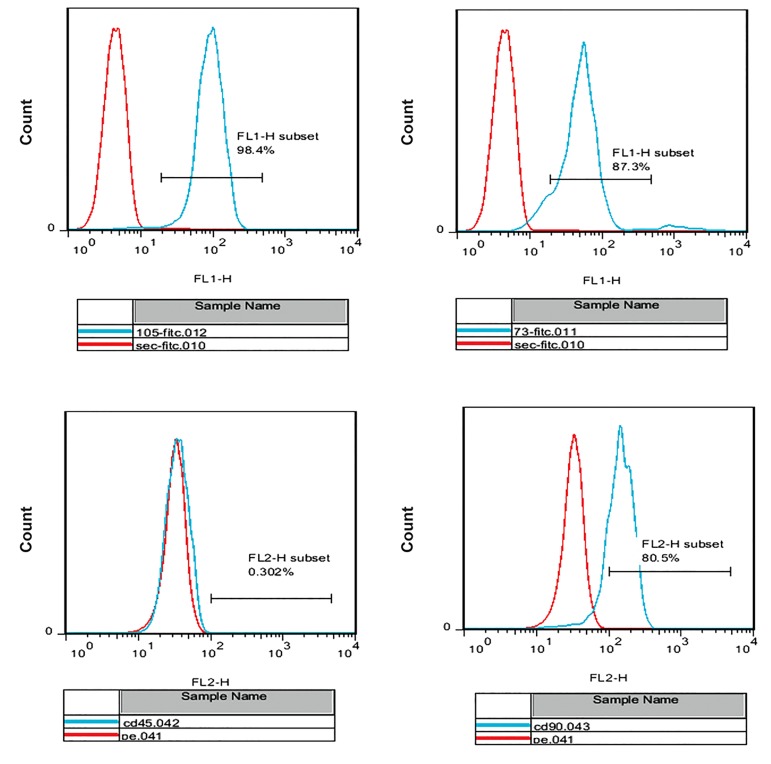
Flow cytometry analysis of adipose-derived stem cell (ADSCs), markers showed positive expressions of 98.4% of ADSCs, CD105^+^, 87.3% cells are CD73^+^, 80.5% are CD90 and 0.303% are CD45 positive.

**Fig.2:**
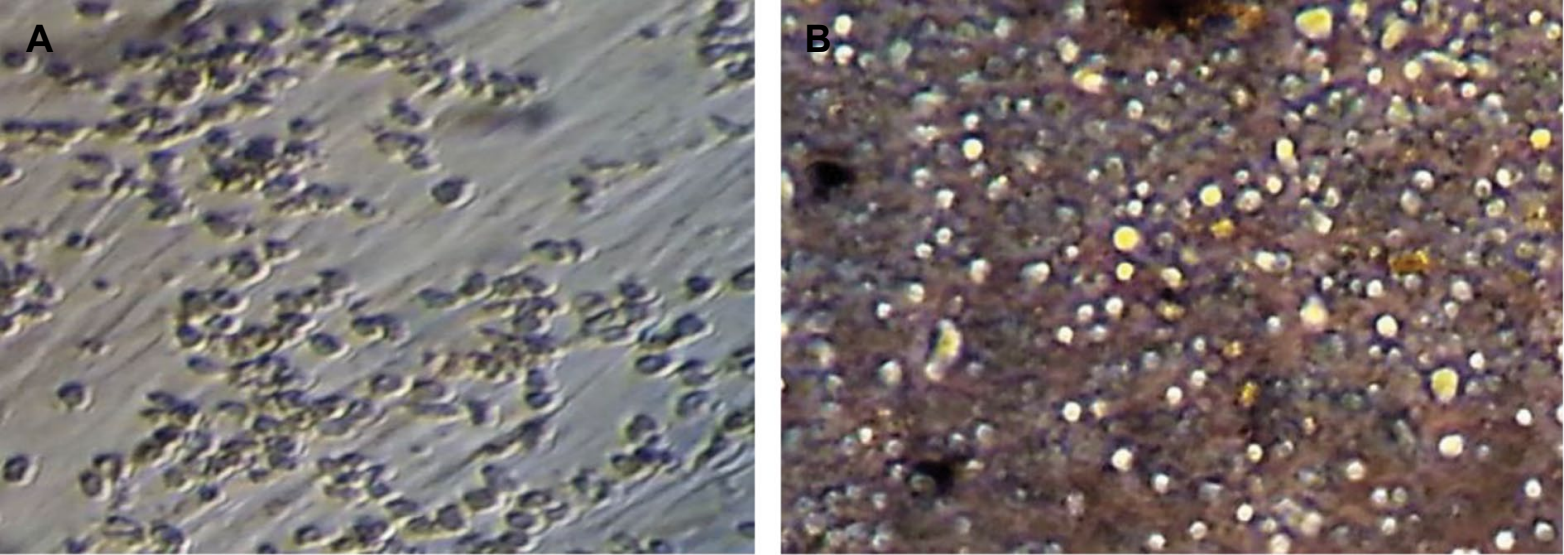
Cultured hematopoietic stem cells on an adipose-derived stem cell feeder layer. A. After 2 days and B. After 7 days.

**Fig.3:**
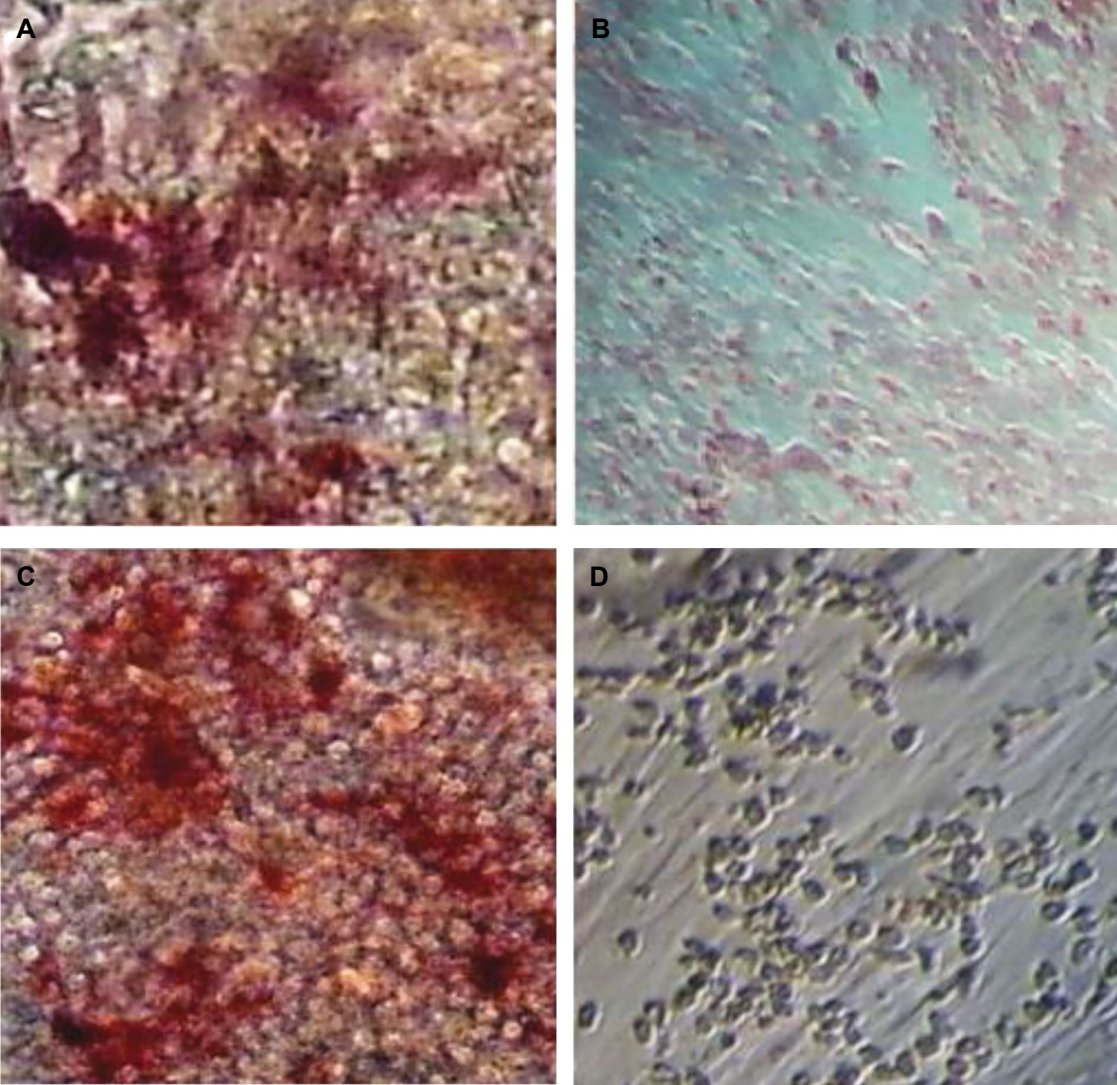
Osteogenic differentiation of adipose-derived stem cell, 200. A. Positive reaction in osteoblastic differentiated cells with alizarin red staining, B. Undifferentiated cells, C. Osteoblast differentiated cells with increased alkaline phosphatase activity, and D. Undifferentiated cells.

We performed flow cytometry analyses of the ADSC surface antigen markers, which resulted inpositive reactions for CD105 (98.4%), CD90 (80.5%), and CD73 (87.3%) antibodies. ADSCs were negative for CD45 (0.302%) (Fig .1). Cultured hematopoietic stem cells on an adipose-derived stem cell feeder layer after 2 and 7 days have shown in Figure 2. We performed alizarin red staining to assess the ability of ADSCs to differentiate osteoblast cells. The results confirmed the osteogenic potential of the ADSCs (Fig .3).

**Fig.4:**
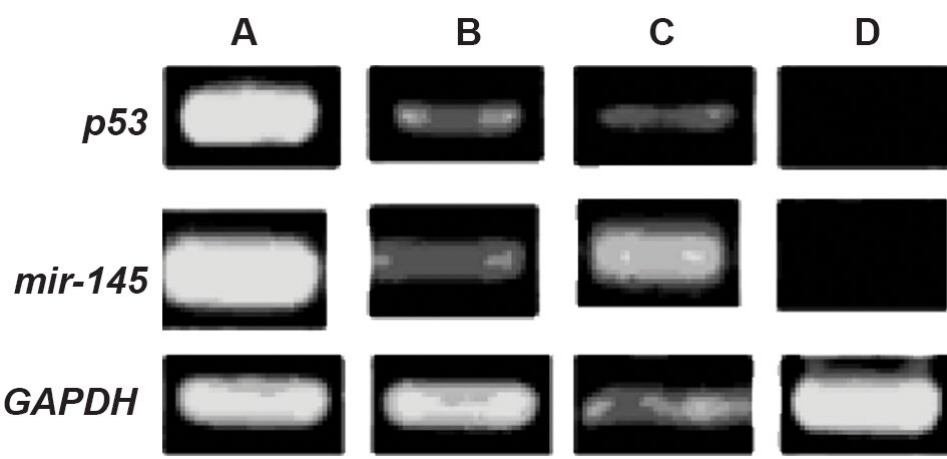
Analysis of *p53* and *miR-145* expressions by reverse transcription PCR in A, B, C, and D groups. A; Fresh CD34^+^ cells, B; CD34^+^ cells cultured in the presence of cytokines, C; CD34^+^ cells indirectly cultured on feeder layer, and D; CD34^+^ cells directly cultured on feeder layer. GAPDH group was considered the control group.

We found that *p53* expressed less than the other groups. Our results showed lower expression of the *p53* gene on the ThinCert™ plate with 0.4 μm pore size compared to HSCs cultured directly on the ADSCs feeder layer. The microporous membrane prevented direct contact between HSCs and the feeder layer. Consequently, there was increased *p53* expression compared to cells that had direct contact with the ADSC feeder layer (Fig .4).

**Fig.5:**
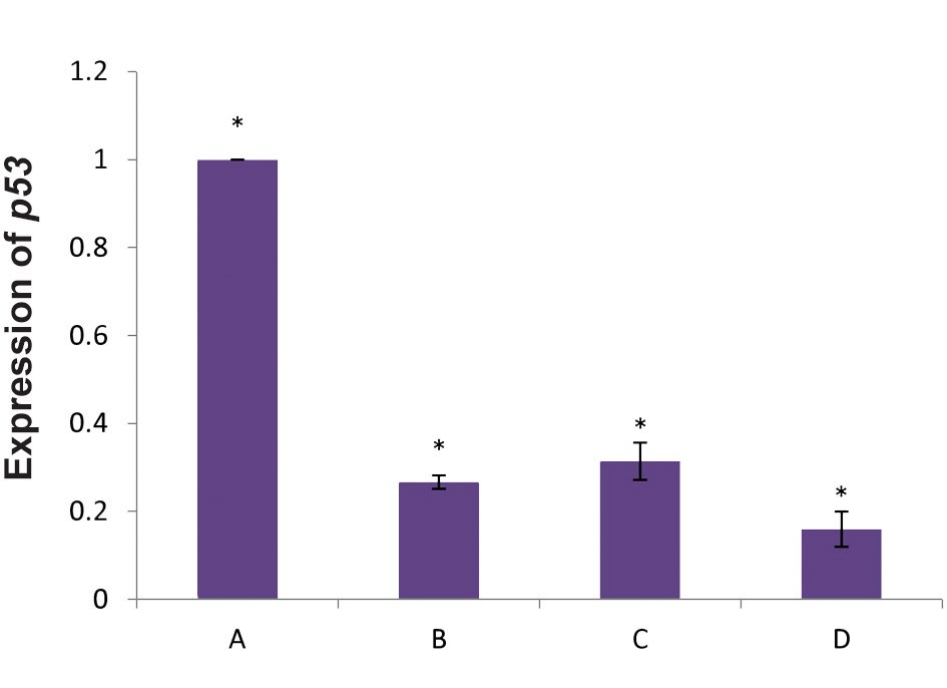
Analysis of *p53* gene expression in fresh CD34^+^ cells by real-time polymerase chain reaction compared to the other groups. A; *p53* gene expression in fresh CD34^+^ cells, B; Expression of *p53* in CD34^+^ cells in the presence of cytokines, C; Expression of *p53* in CD34^+^ cells indirectly cultured on the feeder layer, D; Expression of *p53* in CD34^+^ cells directly cultured on the feeder layer. Fresh CD34^+^ cells showed significant increase in *p53* gene expression compared to the other groups, and *; P<0.05.

**Fig.6:**
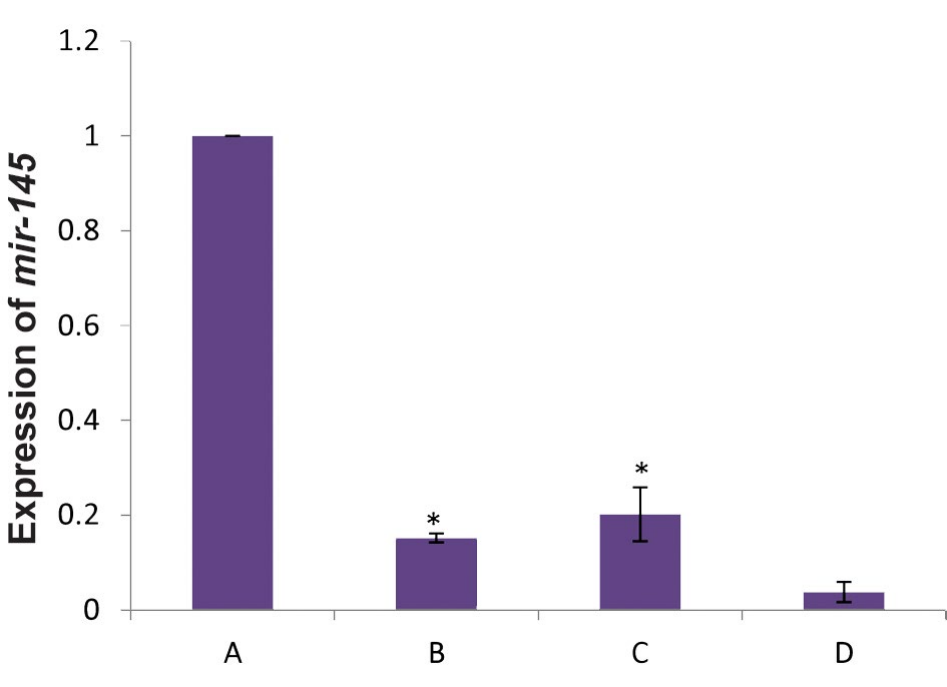
Analysis of *miR-145* expression in fresh CD34^+^ cells by real-time polymerase chain reaction compared to the other groups. A; miR-145 expression in fresh CD34^+^ cells, B; Expression of miR-145 in CD34^+^ cells in the presence of cytokines, C; Expression of miR-145in CD34^+^ cells indirectly cultured on the feeder layer, D; Expression of miR-145 in CD34^+^ cells directly cultured on the feeder layer. Fresh CD34^+^ cells showed significant increase in *miR-145* expression compared to the other groups, and *; P<0.05.

Results of (qRT) PCR analysis were the same as RTPCR analysis. We observed the highest expression of the p53gene in CD34+HSCs (P<0.05). There was lower p53expression in the presence of the ADSC feeder layer compared to the other experiments (P<0.05, Fig .5). Analysis of *miR-145* expression in fresh CD34^+^ cells by real-time polymerase chain reaction compared to the other groups has shown in Figure 6.

## Discussion

Our results have shown that HSCs had higher selfrenewal in the presence of the ADSCs feeder layer compared to the other groups. Because of insufficient numbers of HSCs, expansion of these cells is important for clinical applications. Recently, it was reported that a bone marrow MSC feeder layer along with cytokines such as SCF and TPO increased proliferation of HSCs (2). Glettig and Kaplan (16) reported that different feeder layers for HSCs limited the differentiation of these cells. Our data revealed that the expression of *p53* as a selfrenewal inhibitor gene in HSCs cultured on a feeder layer was lower than the other groups. Tumor suppressor *p53* has been shown to direct regulation of a number of microRNAs such as the miR-34 family and *miR-145 *(17). Sachdeva et al. (13) reported suppression of c-Myc by *p53*-induced *miR-145. miR-145* was reported to inhibit various cancers by targeting several protein coding genes such as c-Myc. *p53* represses c-Myc through induction of the tumor suppressor *miR-145* (14). Suzuki et al. (18) reported that a central tumor suppressor, *p53*, enhanced the post-transcriptional maturation of several microRNAs with growth-suppressive function, including miR-143 and *miR-145*, and miR-16-1.

Suh et al. (19) have found that *miR-145* is regulated by DNA methylation and *p53* gene mutation in some cancers, and *p53* increased the expression level of miR-145. Dong et al. (17) established a new link between *p53* and *miR-145* in tumor growth regulation and metastasis in ovarian carcinoma. There have been no comprehensive studies on the role of microRNAs in HSCs. Our findings showed lower expression levels of *p53* and *miR-145* in HSCs cultured on ADSCs compared to the groups without feeder layers. In terms of the tumor suppressive role of *miR-145* and *p53*, reduced expression of these two genes in the present study indicated that ASDCs could cause growth induction by inhibition of apoptosis. Downregulation of *p53* and consequently miR-145 in HSCs could cause increased proliferation of HSC. On the other hand it has been shown that *miR-145* is induced during differentiation, and it directly silences stem cell self-renewal and pluripotency (20). The results of the present study suggested that suppression of *miR-145* of HSCs cultured on ASCs altered the *p53*-mediated cell cycle arrest.

Our results showed that the expression of miR-145 and *p53* gene on a Thin Cert™ plate with 0.4 μm pore sized groups were lower than HSCs cultured directly on the ASCs feeder layer group. It has been shown that direct contact between HSCs and a feeder layer was critical for expansion of cells (2). da Silva et al. (21) reported that direct contact of HSCs and a feeder layer could increase HSC self-renewal. Alakel et al. (22) showed that direct contact between HSCs and a bone marrow MSCs feeder layer could improve self-renewal of HSCs and can affect migratory behavior of HSCs.

## Conclusion

*miR-145* appears to increase proliferation of HSC cultured on ADSCs by impairing *p53* function. Defining the role of ADSCs in controlling the HSC self-renewal through reduced *miR-145* and *p53* may lead to the treatment and prevention of hematopoietic disorders. Improvement of HSCs self-renewal direct cultured on ADSCs is associated with reduced expression of *miR-145* and *p53*.
